# Quantitative MR-Neurography at 3.0T: Inter-Scanner Reproducibility

**DOI:** 10.3389/fnins.2022.817316

**Published:** 2022-02-16

**Authors:** Fabian Preisner, Rouven Behnisch, Véronique Schwehr, Tim Godel, Daniel Schwarz, Olivia Foesleitner, Philipp Bäumer, Sabine Heiland, Martin Bendszus, Moritz Kronlage

**Affiliations:** ^1^Department of Neuroradiology, Heidelberg University Hospital, Heidelberg, Germany; ^2^Institute of Medical Biometry and Informatics, Heidelberg University, Heidelberg, Germany; ^3^Center for Radiology dia.log, Altötting, Germany

**Keywords:** magnetic resonance imaging, peripheral nervous system, magnetic resonance neurography, biomarkers, reproducibility of results

## Abstract

**Background:**

Quantitative MR-neurography (MRN) is increasingly applied, however, the impact of the MR-scanner on the derived parameters is unknown. Here, we used different 3.0T MR scanners and applied comparable MR-sequences in order to quantify the inter-scanner reproducibility of various MRN parameters of the sciatic nerve.

**Methods:**

Ten healthy volunteers were prospectively examined at three different 3.0T MR scanners and underwent MRN of their sciatic nerve using comparable imaging protocols including diffusion tensor imaging (DTI) and T2 relaxometry. Subsequently, inter-scanner agreement was assessed for seven different parameters by calculating the intraclass correlation coefficients (ICCs) and the standard error of measurement (SEM).

**Results:**

Assessment of inter-scanner reliability revealed good to excellent agreement for T2 (ICC: 0.846) and the quantitative DTI parameters, such as fractional anisotropy (FA) (ICC: 0.876), whereas moderate agreement was observed for proton spin density (PD) (ICC: 0.51). Analysis of variance identified significant inter-scanner differences for several parameters, such as FA (*p* < 0.001; *p* = 0.02), T2 (*p* < 0.01) and PD (*p* = 0.02; *p* < 0.01; *p* = 0.02). Calculated SEM values were mostly within the range of one standard deviation of the absolute mean values, for example 0.033 for FA, 4.12 ms for T2 and 27.8 for PD.

**Conclusion:**

This study quantifies the measurement imprecision for peripheral nerve DTI and T2 relaxometry, which is associated with the use of different MR scanners. The here presented values may serve as an orientation of the possible scanner-associated fluctuations of MRN biomarkers, which can occur under similar conditions.

## Introduction

Magnetic resonance neurography (MRN) has become a valuable technique for evaluation of the peripheral nervous system (PNS) (Filler et al., 1996; Thawait et al., 2011; Chhabra et al., 2018). Morphological nerve imaging, which usually includes high-resolution, T2-weighted (T2w) sequences, has already been established in clinical routine and enables detection of tissue damage on a fascicular level (Pham et al., 2014; Baumer et al., 2016). The diagnostic value of morphological MRN, however, may seem limited since various neuropathies present with the common feature of an elevated T2w-signal, with or without an increase in fascicle caliber (Bäumer et al., 2011; Kronlage et al., 2017a). Quantitative imaging techniques, such as diffusion tensor imaging (DTI) (Baumer et al., 2014; Breckwoldt et al., 2015; Breitenseher et al., 2015) and T2 relaxometry (Kollmer et al., 2015; Vaeggemose et al., 2017a; Kronlage et al., 2019b) might improve the diagnostic performance of MRN by providing additional contrasts and thus potentially pave the way for a formulation of standardized diagnostic criteria.

Diffusion tensor imaging, which has been evaluated in peripheral neuropathies of various etiologies, allows to assess microstructural organization of anisotropic tissues, such as peripheral nerves, and offers four major biomarkers (Hagmann et al., 2006; Mori and Zhang, 2006). The fractional anisotropy (FA) serves as a marker of nervous tissue integrity and technically describes the degree of anisotropy of diffusion being a scalar value between zero (isotropic diffusion) and one (all diffusion in one direction) (Kronlage et al., 2017b; Godel et al., 2019; Kim et al., 2019). While mean diffusivity (MD) characterizes the overall diffusion independent of the direction, axial diffusivity (AD) provides a measure of water diffusion parallel to axonal fiber tracts. Radial diffusivity (RD), on the other hand, quantifies diffusion perpendicular to the principal nerve axis and is considered a biomarker of demyelination (Heckel et al., 2015; Kronlage et al., 2017b).

T2 relaxometry is a quantitative imaging technique that provides an estimate of the transverse relaxation time (T2), and also yields the parameter proton spin density (PD). In contrast to T2, PD is regarded as a semi-quantitative parameter since it is directly dependent on the MR signal and related parameters. T2 relaxometry is commonly based on a multi-echo spin echo (MSE) sequence and fitting of an exponential function (Tofts and du Boulay, 1990; Boulby, 2003). While T2 relaxometry has been extensively studied in the central nervous system, only a few studies have applied it to peripheral nerves with promising results (Kollmer et al., 2015, 2018; Vaeggemose et al., 2017b; Fortanier et al., 2020). In particular, it may allow for a better understanding of pathological mechanisms on a macromolecular level, since T2 reflects free-water protons and PD accounts for total water content including protons bound to macromolecules (Tofts and du Boulay, 1990; MacKay et al., 1994; Tofts, 2003).

Quantitative imaging techniques are increasingly studied in the PNS. While many of them have been proposed to produce valuable MR-biomarkers, they still have not been implemented in clinical routine yet, since it is essential to prove their reliability and reproducibility upon application. Preferably, the measurement error that is expected in different situations should be quantified in order to obtain orientation values regarding the precision of quantitative MRN techniques since the use of different hardware, software and/or readers are known to influence quantitative parameters (Guggenberger et al., 2012, 2013; Preisner et al., 2019, 2021). Furthermore, normative data is dependent on imaging parameters, demographic variables and post-processing algorithms (Chen et al., 2019; Hofstadler et al., 2019; Kronlage et al., 2019b). Recent studies have shown that DTI and T2 relaxometry of peripheral nerves provide reliable results when considering different readers or repetitive scans (Andreisek et al., 2010; Tagliafico et al., 2011; Ho et al., 2017; Preisner et al., 2019, 2021). However, those studies were conducted on identical MR scanners. In a real-world setting a change of the MR scanner is not unlikely, especially in a follow-up of a systemic neuropathy over several years. Moreover, a potential use of quantitative biomarkers as objective criteria for specific neuropathies is only conceivable if the influence of the scanner hardware is only minor. Also, when defining threshold values as diagnostic criteria for certain diseases, it is crucial to know the range of fluctuation, which must be considered, when different scanners are used. While one study reported promising first results for FA and MD measurements using different scanners (Guggenberger et al., 2013), a systematic assessment of inter-scanner reliability of peripheral nerve MRN biomarkers is still lacking.

The purpose of this study was therefore to assess the inter-scanner reliability of sciatic nerve DTI and T2 relaxometry by providing intraclass correlation coefficient (ICC-) and standard error of measurement (SEM-) values, respectively. We prospectively examined a cohort of ten healthy volunteers who each underwent MRN on three different MR scanners.

## Materials and Methods

**FIGURE 1:**
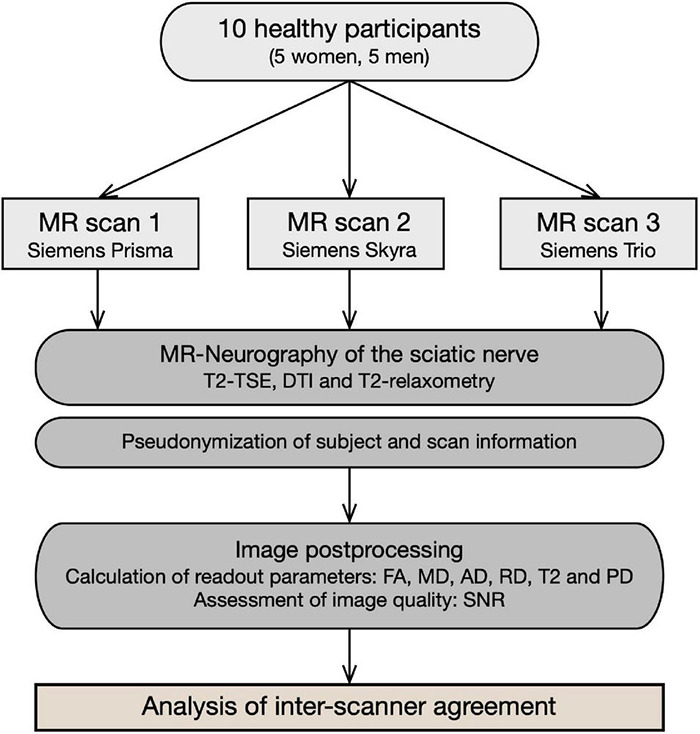
Flowchart of study design. Ten healthy participants underwent repeated multiparametric MR neurography of their sciatic nerve using three different MR scanners. Subsequent image analysis included standardized post-processing algorithms and quantitative assessment of DTI and T2 relaxometry parameters. Finally, inter-scanner agreement was analyzed, and results are expressed in the form of ICC and SEM.

This study was approved by the institutional ethics committee. Written informed consent was obtained from all participants. The study design is summarized in Figure 1.

### Study Subjects

Ten healthy adults (>18 years, 5 men, 5 women) were prospectively enrolled for this study. Mean age was 25.4 ± 1.1 years (range: 24 – 28 years), mean height was 1.73 ± 0.11 m, mean weight was 69.6 ± 19.2 kg and mean BMI was 23 ± 3.9 (range: 18.4 – 29.9). Exclusion criteria were any known or history of peripheral nerve disease as well as general contradictions for MRI.

### MR Imaging

**FIGURE 2:**
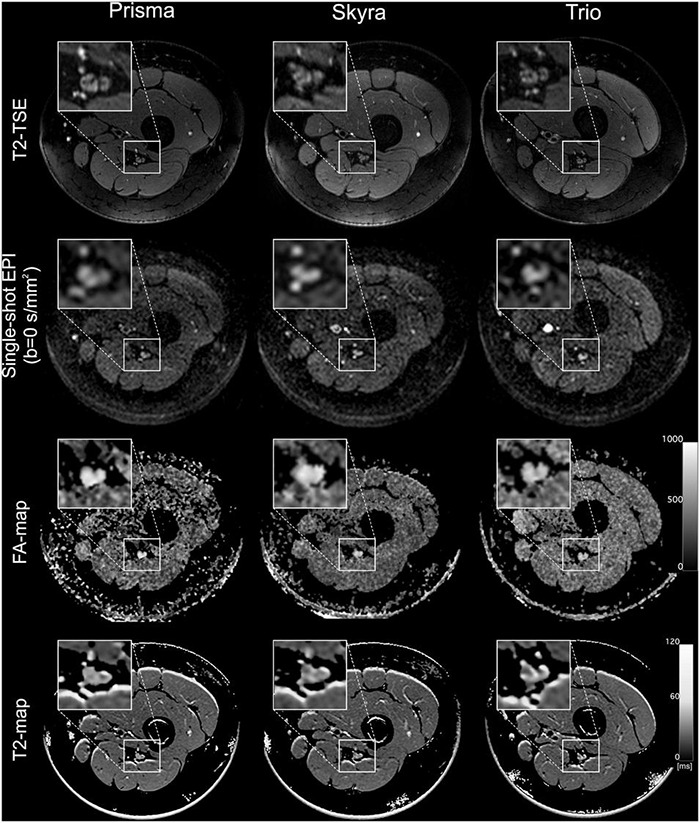
MR imaging of the left leg at mid-thigh level acquired in the same individual on three different MR scanners (Prisma, Skyra, and Trio). From top to bottom, the rows show representative images for a T2 turbo spin echo (TSE) sequence, a single-shot echo planar imaging sequence (b0-image), a calculated FA-map and an exemplary T2-map generated by the OsiriX plugin T2 map. Insets show a magnification of the sciatic nerve.

All participants received three different MR scans of their sciatic nerve using three different MR scanners: (1) Magnetom Prisma-FIT (bore size 60 cm) (Siemens Healthineers, Erlangen, Germany), (2) Magnetom Skyra (bore size 70 cm) (Siemens Healthineers), and (3) Magnetom TIM-TRIO (bore size 60 cm) (Siemens Healthineers). Every scan was performed in supine position with legs extended using a 15-channel transmit-receive knee coil (Siemens Healthineers), which was placed at mid to distal thigh level. The coil was positioned such that its distal end aligned to the distal patella in order to ensure high reproducibility. Additional pads were used to immobilize the thigh and to avoid motion artifacts. Then, MRN protocols were carried out including high-resolution T2-weighted imaging, DTI and T2 relaxometry. Care was taken that parameters determining contrast and geometry were comparable with respect to the different hardware. Detailed sequence parameters are listed in Table 1. Representative MRN images are shown in Figure 2.

**TABLE 1:** MR imaging parameters.

	T2w (turbo spin echo sequence)			Diffusion tensor imaging (single-shot echo planar imaging sequence)			T2 relaxometry (12-echo multi-echo spin echo sequence)		
	Prisma	Skyra	Trio	Prisma	Skyra	Trio	Prisma	Skyra	Trio
Repetition time (TR) [ms]	8,150	8,150	7,000	4,000	4,000	4,000	2,400	2,400	2,400
Echo time (TE) [ms]	54	54	55	83	93	92.8	10, 20…120	10, 20…120	10, 20…120
Field of view (FOV) [mm^2^]	160 × 160	160 × 160	160 × 160	160 × 160	170 × 170	160 × 160	159 × 159	159 × 159	159 × 159
Matrix size	512 × 333	512 × 333	512 × 333	128 × 128	128 × 128	128 × 128	192 × 169	192 × 169	192 × 169
Slice thickness [mm]	3.5	3.5	3.5	4	4	4	3.5	3.5	3.5
Interslice gap [mm]	3.85	3.85	3.85	5.2	5.2	5.2	3.5	3.5	3.5
Number of slices	41	41	41	18	18	18	11	11	11
Fat suppression	Yes	Yes	Yes	Yes	Yes	Yes	Yes	Yes	Yes
Number of averages	2	2	2	3	3	3	1	1	1
Echo train length	13	13	13	59	59	59	12	12	12
Refocusing flip angle [°]	150	150	150	180	180	180	180	180	180
*b*-value 1 [s/mm^2^]	n. a.	n. a.	n. a.	0	0	0	n. a.	n. a.	n. a.
*b*-value 2 [s/mm^2^]	n. a.	n. a.	n. a.	1,000	1,000	1,000	n. a.	n. a.	n. a.
Diffusion encoding directions	n. a.	n. a.	n. a.	21	21	21	n. a.	n. a.	n. a.
Bandwidth (Hz/Pixel)	180	180	181	1,220	1,220	1,395	190	190	190
Acquisition time	3 min 56 s	3 min 56 s	3 min 23 s	4 min 32 s	4 min 32 s	4 min 48 s	6 min 43 s	6 min 43 s	6 min 44 s

### Analysis of Quantitative Magnetic Resonance Neurography

Images were analyzed using the DICOM-viewer OsiriX (Pixmeo Sarl, Switzerland). Initially, image quality was rated as sufficient or insufficient by F.P. (with more than 5 years of experience in neuromuscular imaging) for further analysis. Subsequently, region-of-interest (ROI) based manual nerve segmentation was performed for seven centrally located slices of the image slab using the high-resolution T2-weighted images in which the borders of the nerve were clearly identifiable. Similar approaches have been used in various studies of systemic neuropathies (Kronlage et al., 2017b; Simon et al., 2017; Lichtenstein et al., 2018) and healthy volunteers (Preisner et al., 2019, 2021). To avoid the inclusion of perineurial fat, nerve segmentation was restricted to the tibial portion of the sciatic nerve. All obtained ROIs were then copied to the corresponding b0-image (*b* = 0 s/mm^2^) for DTI analysis and to the corresponding multi-echo spin echo (MSE) slice (*TE*_20ms_) for assessment of T2 relaxometry using the software’s in-built image co-registration tool and, if necessary, manually corrected for distortion artifacts, as described previously (Preisner et al., 2021).

Subsequently, the DTI-derived parameters FA, MD, AD and RD were obtained for each slice separately using the OsiriX plug-in DTI map with a preset for noise threshold of 14 (referring to the voxel signal value in the b0-image [arbitrary units]). T2 relaxometry was conducted using a 12-echo MSE sequence (TE_10–120 *ms*, Δ = 10 *ms*_), of which only the six even echoes (*TE*_20,40,60,80,120 *ms*_) were used for further quantitative analysis. This included a slice-wise determination of the ROI signal intensity using the OsiriX plug-in ROI-enhancement and fitting to a mono-exponential function:


$$S\left(\mathrm{TE}\right)=\text{PD}\times {\text{e}}^{-\frac{\mathrm{TE}}{\mathrm{T2}}}+\mathrm{offset},$$

as described in previous works (Milford et al., 2015; Kronlage et al., 2017a), where S(TE) equals the signal intensity at a given echo time TE, T2 is the transverse relaxation time and PD is a value proportional to proton density per voxel. Moreover, a normalized PD was calculated (further referred to as PD_*Ratio*_) by dividing the PD of the sciatic nerve by a PD of skeletal muscle, the latter of which was assessed by ROI-based measurements in the adjacent musculature (M. semimembranosus or M. adductor magnus). After slice-wise calculation of DTI parameters, T2 and PD, all parameters were averaged over all seven slices for further analysis.

### Quantitative Assessment of Image Quality

The signal-to-noise ratio (SNR) is commonly reported to describe image quality. To determine SNR_*DTI*_, we used a “five-region approach” in the b0-image. Therefore, a total number of four ROIs with identical size (4 cm^2^) were positioned in the corners of the background and the standard deviations of the background signals were averaged over all four ROIs to calculate “noise.” Nerve signal intensity was subsequently divided by noise to calculate SNR_*DTI*_ (Supplementary Figure 1). This was performed on all seven slices, which were used for further DTI-analysis, and SNR values then were averaged over all slices. SNR of T2 relaxometry was assessed similarly using the MSE sequence (*TE*_10ms_).

### Statistical Analysis

Statistical testing was performed using SPSS (Version 24; SPSS Inc.) and R (Version 4.0.3; R Foundation for Statistical Computing). Graphs were created using GraphPad Prism (Version 9.0.2; GraphPad Software Inc.).

Descriptive statistics include mean values, standard deviation, interquartile range, and minimum to maximum values for every quantitative MRN parameter. One-way analysis of variance with pairwise comparisons was conducted to test for differences between scanners and Bonferroni correction was applied to correct for multiplicity. To assess inter-scanner agreement, a two-way mixed effects model, ICC (3,1) according to Shrout and Fleiss, was applied and ICCs with 95% confidence intervals (CIs) were calculated (Shrout and Fleiss, 1979). According to Koo and Li, ICC values between 0.5 and 0.75, between 0.75 and 0.9, and greater 0.9 were regarded as indicative for moderate, good and excellent agreement (Koo and Li, 2016). Additionally, mean absolute percentage errors between MR scanners were calculated for each parameter and participant and subsequently averaged over all participants, respectively. In this context, the mean value between the three scans served as the accepted true value for each parameter. Furthermore, Bland-Altman analyses for FA, T2, PD and PD_*Ratio*_ were calculated and measurement bias with 95% CIs as well as upper and lower limits of agreements are reported. Measurement distribution within the limits of agreement is visualized in Bland-Altman plots. *P*-values ≤ 0.05 were regarded as statistically significant.

## Results

### Descriptive Statistics

**FIGURE 3:**
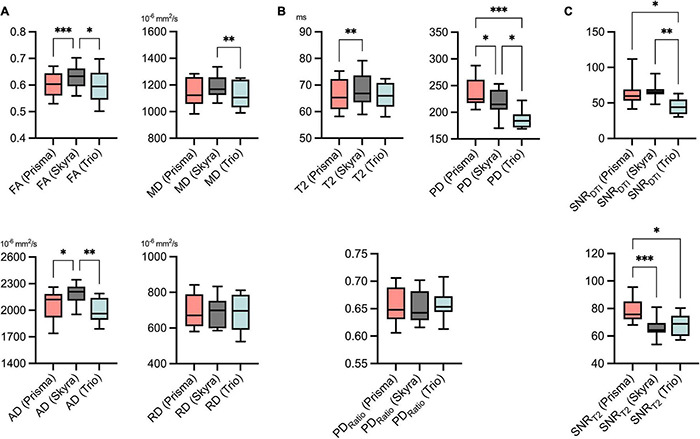
Descriptive statistics for all DTI **(A)** and T2 relaxometry **(B)** parameters, SNR **(C)** values and all three scans, respectively. SNR was assessed separately for diffusion tensor imaging (SNR_*DTI*_) and the T2-relaxometry sequence (SNR_*T2*_). Values are illustrated as boxplots to visualize measurement distribution. FA, fractional anisotropy; MD, mean diffusivity; AD, axial diffusivity; RD, radial diffusivity; T2, transverse relaxation time; PD, proton spin density [proportional to proton density per voxel]; SNR, signal-to-noise ratio. Statistical significance is indicated on a level of *p ≤ 0.05, **p ≤ 0.01, and ***p ≤ 0.001.

Detailed descriptive statistics for all parameters and MR scans are shown in Figure 3 and Supplementary Table 1. The overall mean value and standard deviation averaged over all MR scanners was 0.61 ± 0.05 for FA, 1152.8 ± 98.9 × 10^–6^ mm^2^/s for MD, 2076.2 ± 161.7 × 10^–6^ mm^2^/s for AD, 690.6 ± 96 × 10^–6^ mm^2^/s for RD, 66.8 ± 5.9 ms for T2, 213 ± 30.1 for PD and 0.66 ± 0.03 for PD_*Ratio*_.

Comparison of mean values of the different MR scanners by ANOVA showed predominantly small but statistically significant differences for FA (Prisma vs. Skyra: 0.601 vs. 0.632, *p* < 0.001; Skyra vs. Trio: 0.632 vs. 0.597, *p* = 0.022), for MD (Skyra vs. Trio: 1,189 vs. 1,121 × 10^–6^ mm^2^/s, *p* < 0.01), for AD (Prisma vs. Skyra: 2,054 vs. 2,182 × 10^–6^ mm^2^/s, *p* = 0.016; Skyra vs. Trio: 2,182 vs. 1,993 × 10^–6^ mm^2^/s, *p* < 0.01), for T2 (Prisma vs. Skyra: 66.2 vs. 68.3 ms, *p* < 0.01) and for PD (Prisma vs. Skyra: 235 vs. 217, *p* = 0.022; Prisma vs. Trio: 235 vs. 187, *p* < 0.001; Skyra vs. Trio: 217 vs. 187, *p* = 0.016). No significant differences could be detected for RD and PD_*Ratio*_.

### Inter-Scanner Agreement

Assessment of inter-scanner reliability showed good agreement for FA, MD and T2 with ICCs ranging from 0.83 for MD to 0.88 for FA (all ICC values in Table 2). Excellent agreement was observed for RD with an ICC of 0.92. Inter-scanner reliability was moderate regarding AD, PD and PD_*Ratio*_ with ICC values ranging from 0.51 for PD to 0.7 for AD.

**TABLE 2:** Intraclass correlation coefficients (ICCs) and the standard error of measurement (SEM).

Variables	ICC (3,1)	95% CI	SEM	Mean absolute percentage error [%]
FA	0.876	[0.687, 0.964]	0.033	4.37
MD	0.828	[0.588, 0.949]	70.46 [10^–6^ mm^2^/s]	4.47
AD	0.697	[0.361, 0.904]	138.8 [10^–6^ mm^2^/s]	5.85
RD	0.915	[0.776, 0.976]	50.06 [10^–6^ mm^2^/s]	4.35
T2	0.846	[0.624, 0.955]	4.12 [ms]	3.48
PD	0.51	[0.119, 0.826]	27.8^†^	11.12
PD ratio	0.635	[0.272, 0.88]	0.03	2.39

*Intraclass correlation coefficients (ICC) with 95% confidence intervals (CI) were calculated according to Shrout and Fleiss. Calculation of SEM values is based on Popovic and Thomas (2017). FA, fractional anisotropy; MD, mean diffusivity; AD, axial diffusivity; RD, radial diffusivity; T2, transverse relaxation time; PD, proton spin density;*

^†^ *proportional to proton density per voxel.*

Calculated SEM values were mostly within the observed standard deviation of the overall mean values, for example SEM was 0.033 for FA, 4.12 ms for T2 and 27.8 for PD. SEM values for all parameters as well as mean absolute percentage errors are listed in Table 2.

Bland-Altman analysis is shown in Table 3 and Supplementary Figure 2. Maximal measurement bias between two MR scanners was 0.035 for FA, 67.9 × 10^–6^ mm^2^/s for MD, 188.8 × 10^–6^ mm^2^/s for AD, 11.7 × 10^–6^ mm^2^/s for RD, 2.4 ms for T2, 48.44 for PD and 0.003 for PD_Ratio_.

**TABLE 3:** Bland-Altman analyses for inter-scanner agreement.

Variables	Prisma vs. Skyra	Skyra vs. Trio	Prisma vs. Trio
**FA**			
bias	−0.031	0.035	0.004
sd of bias	0.015	0.032	0.029
lloa of 95% CI	−0.061	−0.028	−0.053
uloa of 95% CI	−0.001	0.098	0.061
**MD [10^–6^ mm^2^/s]**			
bias	−41	67.9	26.93
sd of bias	64	48.68	58.83
lloa of 95% CI	−166	−27.51	−88.39
uloa of 95% CI	84.5	163.3	142.2
**AD [10^–6^ mm^2^/s]**			
bias	−127.7	188.8	61.04
sd of bias	11.07	121.8	107.1
lloa of 95% CI	−344.6	−49.95	−148.8
uloa of 95% CI	89.15	427.5	270.9
**RD [10^–6^ mm^2^/s]**			
bias	4.47	7.2	11.67
sd of bias	39.77	38.43	47.75
lloa of 95% CI	−73.48	−68.12	−81.92
uloa of 95% CI	−82.42	82.52	105.3
**T2 [ms]**			
bias	−2.11	2.42	0.31
sd of bias	1.5	4.29	3.65
lloa of 95% CI	−5.05	−5.99	−6.84
uloa of 95% CI	0.82	10.83	7.46
**PD**			
bias	17.92	30.52	48.44
sd of bias	16.41	26.35	24.13
lloa of 95% CI	−14.25	−21.13	1.15
uloa of 95% CI	50.09	82.17	95.73
**PD ratio**			
bias	0.001	−0.003	−0.002
sd of bias	0.018	0.028	0.032
lloa of 95% CI	−0.034	−0.057	−0.063
uloa of 95% CI	0.036	0.051	0.06

*FA, fractional anisotropy; MD, mean diffusivity; AD, axial diffusivity; RD, radial diffusivity; T2, transverse relaxation time; PD, proton spin density; sd, standard deviation; lloa, lower limit of agreement; uloa, upper limit of agreement; CI, confidence intervals.*

### Signal-to-Noise Ratio

Mean SNR values were calculated for DTI (SNR_DTI_) and the T2 relaxometry sequence (SNR_T2_) for all three MR scanners, respectively. Analyses of variance showed that SNR_DTI_ was significantly higher for Prisma and Skyra compared to Trio (Prisma vs. Trio, *p* = 0.03; Skyra vs. Trio, *p* = 0.002). SNR_T2_ was significantly higher for Prisma compared to Skyra and Trio (Prisma vs. Skyra, *p* = 0.001; Prisma vs. Trio, *p* = 0.027) (Figure 3 and Table 4).

**TABLE 4:** Quantitative signal-to-noise ratio (SNR) analysis for the diffusion tensor imaging (DTI) and T2 relaxometry sequence.

Variables	Prisma (*N* = 10)	Skyra (*N* = 10)	Trio (*N* = 10)	Prisma vs. Skyra	Skyra vs. Trio	Prisma vs. Trio
**SNR (DTI)**						
mean	64	66.5	45.2	*p* = 0.99	*p* = 0.002	*p* = 0.03
standard deviation	20.7	10.9	11.1			
**SNR (T2)**						
mean	78.5	65.9	68.11	*p* = 0.001	*p* = 0.99	*p* = 0.027
standard deviation	8.6	7.4	7.9			

*Calculated p values are displayed as results from analysis of variance with pairwise comparisons and Bonferroni correction.*

## Discussion

This study evaluated the reproducibility of peripheral nerve DTI and T2 relaxometry in different MR scanners at the same field strength. We examined a healthy cohort using three different MR scanners (all 3.0T) and quantified measurement accuracy by reporting ICC- and SEM values for seven different parameters. As a principal finding, differences of some DTI and T2 relaxometry parameters were statistically significant between scanners. In order to provide a measure that allows to estimate the inaccuracy attributed to a change of the MR scanner in an individual patient follow-up, we report the standard error of measurement (SEM) for each parameter.

The authors are aware of only one study by Guggenberger et al. (2013) that has systematically assessed the agreement of FA and apparent diffusion coefficient (ADC) values of the median nerve using three different MR scanners. Similar to that study, we observed that quantitative parameters, such as FA, can differ significantly between different MR scanners. This may in part result from differences in SNR (Figure 3) and the fact that noise plays a role as a systematic source of error when calculating quantitative parameters. Also, factors that are not transparent to the user, such as correction or interpolation processes during image acquisition, may lead to systematic differences in quantitative MRN values. PD was the parameter with the highest inter-scanner variation, as we expected due to the parameter’s dependency on technical properties, such as RF coil, and signal attenuation. Thus, absolute PD values should always be interpreted carefully. Normalizing PD to adjacent muscle tissue has shown to be a more robust parameter and can be used to improve comparability between different scanners, yet accompanying muscular changes should always be considered when observing systemic neuropathies (Kronlage et al., 2019a).

Like in many reliability studies, Guggenberger et al. expressed their results by reporting the ICC, which is a commonly used parameter to describe the reliability of measurements and ranges between 0 and 1. Although the ICC is a useful statistical measure, it should be interpreted with care since different forms of ICC exist and results may vary depending on the selected form, even if applied to the same data (Koo and Li, 2016). Additionally, ICC values can be affected by several factors, such as data range, which means that a higher ICC value does not necessarily indicate less variability (Stratford and Goldsmith, 1997; Lee et al., 2012). In light of these limitations, we here provide the standard error of measurement (SEM) for every parameter in addition to ICC values. The SEM estimates measurement precision independently of the sample variance and is expressed in the same physical unit as the measured quantity, thereby providing a more useful framework for decision making in clinical practice (Popovic and Thomas, 2017).

The expected measurement error, which is associated with different readers (interreader) and repeated scans (test-retest) without switching between different MR scanners, has been estimated in recent studies and corresponding SEM values have been calculated for various quantitative MRN parameters (Preisner et al., 2019, 2021). The SEM values observed in our study, which accounts for the use of different MR scanners, demonstrate a slightly higher measurement error compared to interreader and test-retest observations with one particular MR scanner. For example, we report an SEM for FA considering examinations on different MR scanners of 0.033. In contrast, a repeated MR examination on the same scanner or a change of the reader have been described by SEM values of 0.02, respectively (Preisner et al., 2019). Furthermore, we calculate a measurement error for T2 of SEM = 4.1 ms when using different MR scanners. This value may be compared to a previously reported SEM of 2.7 ms for repeated measurements on the exact same MR scanner (Preisner et al., 2021).

This observation becomes even more relevant when calculating the minimum detectable difference (MDD, equals 2.8 × SEM), which can help to decide whether an observed difference may likely be attributed measurement error, or whether it really indicates a change in the true value (Popovic and Thomas, 2017). If a measured difference is larger than the MDD, there is high certainty that it is due to a change in the true value, e.g., reflecting a substantial change in tissue physiology. As the MDD increases along with SEM and thus a change of the MR scanner, it becomes evident that a greater difference in values will be required to confidently distinguish healthy from diseased nerves when using different MR scanners.

For example, the MDD for FA associated with a change of MR scanner is 2.8 × 0.033 = 0.092. Differences in sciatic nerve FA values between patients and healthy participants have been previously reported and ranged between 0.06 and 0.25 (Mathys et al., 2013; Bernabeu et al., 2016; Markvardsen et al., 2016; Vaeggemose et al., 2017a; Kim et al., 2019). While these differences were statistically significant on a group level, some of these differences, in a theoretical setting and on an individual patient level, would be lower than the calculated MDD and thus not reliably distinguishable from variations due to measurement error.

On a group level, however, these differences may have a lesser impact. The systematic difference between two scanners is reflected by the measurement bias observed in our study. For example, maximal measurement bias for FA was 0.035, which is smaller compared to previously reported differences in patients and healthy participants (see above) and within the standard range of the overall mean values. Similar observations can be made for other DTI parameters as well as T2 relaxometry. However, this systematic bias should be considered, especially when participants of particular study groups are examined at different MR scanners, since this bias alone may lead to statistically significant results.

Taken together, our results concerning the use of different MR scanners produced a higher measurement error compared to recently published measurement errors for repeated scans on the same scanner or a change of reader (Preisner et al., 2019, 2021). This becomes relevant regarding the use of quantitative MRN techniques as biomarkers, since their potential would be limited for individual follow-up examinations, especially when expected differences are subtle, e.g., in cases of peripheral nerve trauma or longitudinal observation of diffusion parameters, where minor changes may reflect fiber organization or myelin sheath integrity (Mathys et al., 2013). Using different MR scanners for larger group studies, however, may in certain situations be justifiable since overall differences – despite in part statistically significant – are not expected to substantially impact the differentiation between healthy and diseased nerves. In this context, however, it is also important to note that statistical significance does not necessarily imply clinical relevance, since small and non-significant differences can be clinically relevant and vice versa. Also, statistical significance is dependent of the power of the test. Due to our study design, we chose an ANOVA with pairwise comparisons, which has a higher power compared to a non-paired test. Therefore, we would like to focus not only on statistical significance but would like to emphasize the absolute amount of this systematic bias, which we quantified in this study, and which may aid as an orientation in future situations when a change of scanner hardware occurs.

There are limitations to this study. First, ten healthy volunteers were included. A larger cohort as well as the inclusion of patients with peripheral nerve disease would allow for optimal variability assessment of quantitative MRN and improve interpretation of measurement fluctuations in relation to inter-scanner differences. The fact that we only used MR scanners from one vendor can be regarded as another limitation. MR scanners from different vendors might introduce a greater variability in hardware and sequence parameters are expected to vary more substantially between different vendors, e.g., regarding RF pulse shape and gradient ramping. Thus, using MR scanners from more than one vendor may have led to higher measurement variability. Minor differences in DTI sequence parameters regarding field of view, echo time and pixel bandwidth may also contribute to the here reported discrepancies in SNR and affect the determined measurement error of DTI parameters, which must be regarded as another relevant limitation of this study. Especially minor variations regarding the field of view with identical matrix size led to different voxel sizes between MR scanners. Taken this into account, we would expect an advantage in SNR_*DTI*_ for Skyra of approximately 13%, but only a 4% higher SNR_*DTI*_ was observed. This, in turn, may be a consequence of hardware differences between the two MR scanners, since Prisma has a smaller bore size and allowed for lower TE values due to a stronger gradient system. Furthermore, SNR calculations were conducted using ROI-based measurements in separate signal and noise regions, although it is known that the use of multi-channel coils and reconstruction filters can lead to over- or underestimation of SNR when using such methods (Dietrich et al., 2007). Like in many *in vivo* studies, an approach based on repeated acquisition to calculate the SNR was not considered feasible with respect to the acquisition time and potential motion artifacts. As a compromise, we used a “five-region approach,” calculated the standard deviation of background noise instead of mean values, and averaged over multiple slices to compensate for inhomogeneous spatial distribution of noise. However, a certain bias is to be expected with this method, which should be considered another limitation. Moreover, we used one vendor-independent post-processing method for analysis of all scans. This allowed to minimize systematic differences resulting during post-processing and helped to attribute the observed differences to the acquisition and processing stages. Furthermore, we focused on the sciatic nerve since it is still the most commonly examined nerve in MRN and most suitable due to its straight course and great caliber. An inclusion of small caliber nerves, for example at the upper extremity, may have led to a higher measurement variability. However, a recent study suggests that DTI- and in particular FA-values of the sciatic nerve can be considered as an objective parameter for the structural integrity of the entire PNS in diabetic neuropathy (Jende et al., 2021). Thus, quantitative MRN of the sciatic nerve seems conceivable in follow-up of systemic neuropathies, although this concept still must be evaluated for other forms of systemic neuropathies in future studies. Lastly, we chose a manual nerve segmentation approach, since it is regarded a well-established method in MRN and proven to result in reliable and reproducible values, both between different readers and scans (Preisner et al., 2019, 2021). In the future, automatic segmentation methods may also become implemented into clinical practice (Balsiger et al., 2018).

In summary, this study quantifies the measurement imprecision for peripheral nerve DTI and T2 relaxometry, which is associated with the use of different MR scanners. The here presented values may serve as an orientation of the possible scanner-associated fluctuations of MRN biomarkers, which can occur under similar conditions.

## Data Availability Statement

The raw data supporting the conclusions of this article will be made available by the authors, without undue reservation.

## Ethics Statement

The studies involving human participants were reviewed and approved by the Ethics Committee of the Medical Faculty of the University of Heidelberg. The patients/participants provided their written informed consent to participate in this study.

## Author Contributions

MK, PB, FP, and MB designed and coordinated the study. VS organized the participants. VS and MK collected the MR data. FP, MK, TG, OF, DS, and SH performed image post-processing and analysed the data. RB performed the main statistical analysis. FP and MK wrote the manuscript with input from all co-authors.

## Conflict of Interest

The authors declare that the research was conducted in the absence of any commercial or financial relationships that could be construed as a potential conflict of interest.

## Publisher’s Note

All claims expressed in this article are solely those of the authors and do not necessarily represent those of their affiliated organizations, or those of the publisher, the editors and the reviewers. Any product that may be evaluated in this article, or claim that may be made by its manufacturer, is not guaranteed or endorsed by the publisher.
