# Supplementary material for: Quantitative MR-Neurography at 3.0T: Inter-Scanner Reproducibility
Source: Front Neurosci. 2022 Feb 16;16:817316. doi: 10.3389/fnins.2022.817316 (PMC8888927; doi:10.3389/fnins.2022.817316)
Supplement: Supplementary file 1 [file Data_Sheet_1.docx]

Supplementary Material

**Supplementary Table 1** Descriptive statistics.

| Variables | Prisma  (N=10) | Skyra  (N=10) | Trio  (N=10) |
| --- | --- | --- | --- |
| FA  mean  standard deviation  Q1 – Q3  min – max | 0.601  0.049  0.571 – 0.643  0.53 – 0.671 | 0.632  0.045  0.599 – 0.654  0.559 – 0.702 | 0.597  0.064  0.553 – 0.636  0.501 – 0.698 |
| MD [10^-6^ mm^2^/s]  mean  standard deviation  Q1 – Q3  min – max | 1148.1  108  1060 – 1256  983 – 1284 | 1189.1  84.6  1128 – 1252  1064 – 1336 | 1121.2  100.5  1041 – 1239  991 – 1252 |
| AD [10^-6^ mm^2^/s]  mean  standard deviation  Q1 – Q3  min – max | 2054  169.9  1954 – 2179  1739 – 2261 | 2181.7  125.3  2147 – 2252  1953 – 2343 | 1993  137.9  1908 – 2132  1790 – 2188 |
| RD [10^-6^ mm^2^/s]  mean  standard deviation  Q1 – Q3  min – max | 696  98.5  613 – 787  580 – 842 | 691.5  85.3  600 – 752  586 – 833 | 684.3  112.4  606 – 778  524 – 812 |
| T2 [ms]  mean  standard deviation  Q1 – Q3  min – max | 66.2  6.2  62 – 71  58.2 – 75.1 | 68.3  6.7  64 – 72  58.9 – 79.2 | 65.8  5.2  63 – 70  58.1 – 72.4 |
| PD  mean  standard deviation  Q1 – Q3  min – max | 235.1  26.7  218 – 259  205 – 288 | 217.2  24.6  208 – 241  170 – 253 | 186.6  16.1  172 – 196  169 – 222 |
| PD ratio  mean  standard deviation  Q1 – Q3  min – max | 0.66  0.032  0.63 – 0.69  0.61 – 0.71 | 0.65  0.029  0.63 – 0.68  0.62 – 0.7 | 0.66  0.027  0.65 – 0.67  0.61 – 0.71 |

FA, fractional anisotropy; MD, mean diffusivity; AD, axial diffusivity; RD, radial diffusivity; T2, transverse relaxation time; PD, proton spin density [proportional to proton density per voxel].


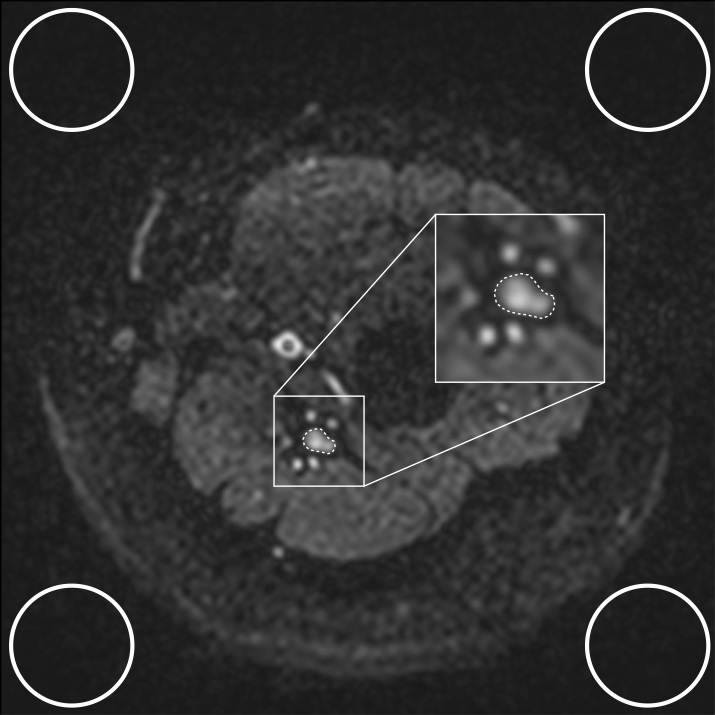


**Supplementary Figure 1.** Determination of SNR_DTI_ using ROI-based measurements in separate signal and noise regions. To determine “noise”, a total number of four ROIs were positioned in the corners of the background in the b0-image to compensate for inhomogeneous spatial distribution of noise, and the standard deviations of the background signals were averaged. Signal of interest (ROI_nerve_) was subsequently divided by noise to calculate SNR. Measurements were performed on seven consecutive slices and subsequently averaged over all slices.

**
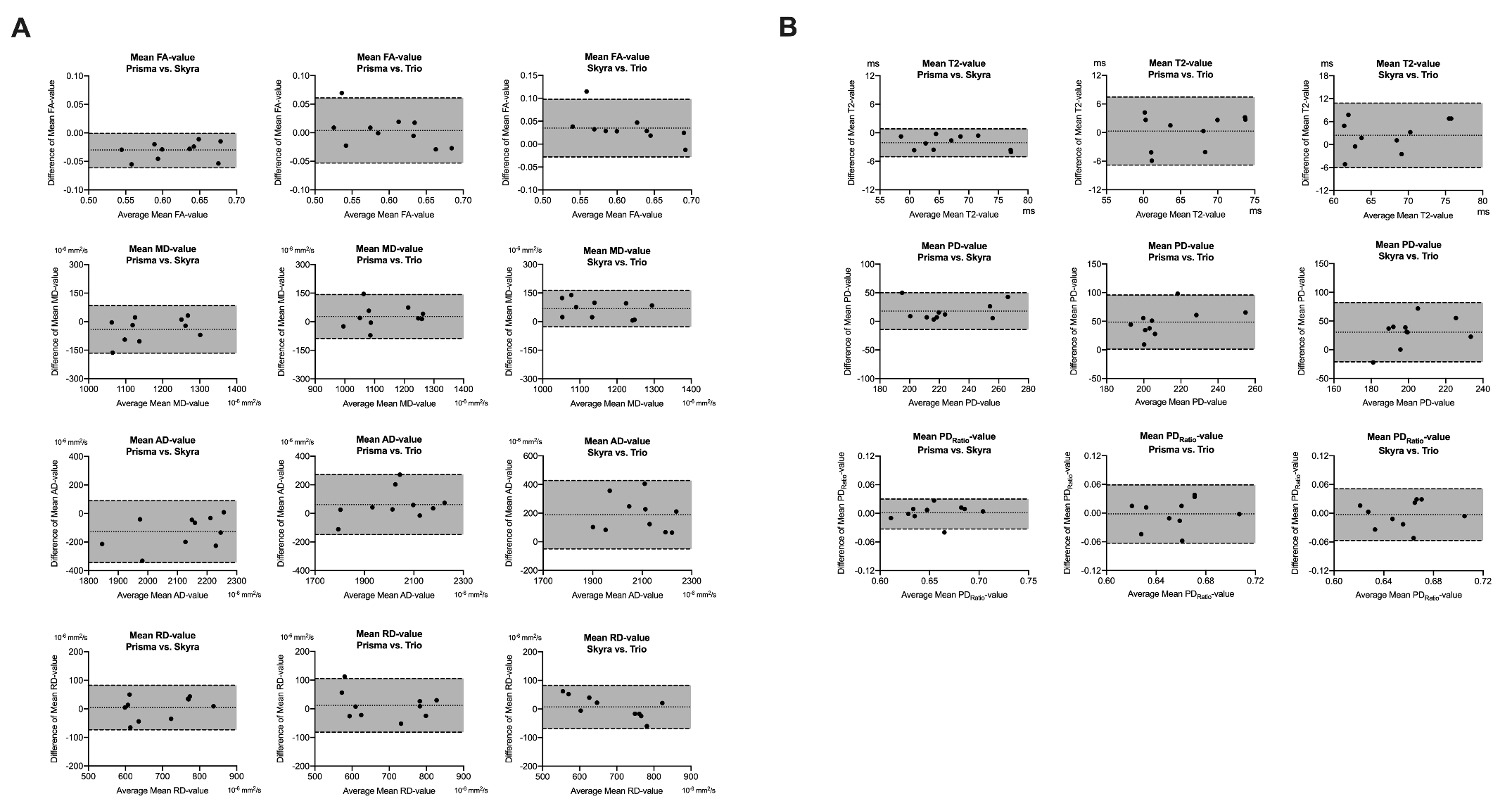
Supplementary Figure 2.** Bland-Altman plots for assessment of inter-scanner agreement of every quantitative readout parameter based on DTI (A) and T2 relaxometry (B). The black dotted line represents the mean of all differences (bias), dashed lines show the 95% limits of agreement. FA, fractional anisotropy; MD, mean diffusivity; AD, axial diffusivity; RD, radial diffusivity; T2, transverse relaxation time; PD, proton spin density [proportional to proton density per voxel].
